# Evaluating UV Stability of *Miscanthus × giganteus* Particles via Radiografting of UV Absorbers

**DOI:** 10.3390/molecules30173649

**Published:** 2025-09-08

**Authors:** Roland El Hage, Dominique Lafon-Pham, Rodolphe Sonnier

**Affiliations:** 1 PCH, IMT–Mines Alès, 6, Avenue de Clavières, 30100 Alès, France; rodolphe.sonnier@mines-ales.fr; 2 EuroMov Digital Health in Motion, University of Montpellier, IMT Mines Ales, 6 Avenue de Clavières, 30319 Alès, France; dominique.lafon@mines-ales.fr

**Keywords:** *Miscanthus × giganteus*, UV absorbers, radiation (*e*-beam)-induced grafting, accelerated weathering, photodegradation, colorimetric analysis

## Abstract

*Miscanthus × giganteus* particles possess excellent advantages in biodegradability and sustainability. However, their susceptibility to ultraviolet (UV) degradation limits wider outdoor applications. In the present work, electron beam (*e*-beam) radiation-induced grafting was used for the first time to attempt covalent grafting of UV absorbers onto miscanthus particles to address a major challenge in natural fiber stabilization. Two UV absorbers, 2-hydroxy-4-(methacryloyloxy) benzophenone (HMB) and 2-(4-benzoyl-3-hydroxyphenoxy) ethyl acrylate (BHEA), were explored using both pre-irradiation and simultaneous approaches. Pre-irradiation grafting did not achieve useful covalent fixation of HMB or BHEA, due in part to the premature decay of radicals at elevated temperatures and with solvent use, and the lignin-based quenching of radicals. Solvent-free mutual irradiation grafting failed due to immobility of the UV absorbers, while grafting of HMB in solvent failed due to radical-scavenging behavior. Grafting of BHEA was successfully achieved under solvent-based simultaneous irradiation, reaching up to 38 wt % DG in a butanone/2.5% H_2_SO_4_ system. This condition led to the improved UV stability of miscanthus particles, in which color change was reduced significantly after 1000 h of accelerated weathering; this was mainly linked to a beneficial pre-darkening effect which was induced by the presence of the acid. This work proposes a route of grafting strategy that aims to improve the photostability of miscanthus particles, paving the way for durable bio-based materials in outdoor composite applications.

## 1. Introduction

UV aging enhancement in natural fibers is of vital importance in prolonging the lifespan and improving the functionality of materials that find utility in various applications, especially among biocomposites [1,2,3,4]. Among the different natural biomasses, *Miscanthus × giganteus* is a sustainable biomass grass of growing cultural and commercial importance. Even though it requires water sufficient to establish the deep roots of newly planted rhizomes [5,6], this noninvasive, sterile C4 crop composed of lignin, cellulose, hemicelluloses, extractives and inorganics [7,8,9,10,11] becomes more water-efficient once established, while also offering advantages in sustainability and biodegradability [12]. Its majority composition of cellulose (>50%) makes miscanthus a promising feedstock for biofuels, paper and fiber-composite materials [13]. It can be cultivated in most climatic zones [14], growing over 3 m tall annually with minimal herbicide and nitrogen requirements. With a high productivity rate, *Miscanthus × giganteus* can produce up to 30 tons of dry matter per hectare. Additionally, pests and diseases have been found to have no significant impact on its cultivation [15]. It should be noted that miscanthus planting areas are still modest and have reached only about 10^4^ ha in the European Union. This situation is linked to establishment costs and market factors. Yet, policy incentives and its strong carbon-sequestration benefits are driving interest in upscaling miscanthus production [16].

However, lignocellulosic materials such as miscanthus or other natural plant fibers are vulnerable to ultraviolet (UV) light degradation, and this can present a significant obstacle. This consideration is essential when the materials are used outdoors, and they are exposed to sunlight, causing the fibers to be altered. In a recent work, we have investigated the UV artificial weathering of *Miscanthus × giganteus* [17]. That study demonstrated alterations in both color and chemical composition throughout the weathering process under ultraviolet (UV) light, along with a limiting effect attributed to the presence of extractives. The study highlights a browning effect occurring after UV weathering, which seems to be associated with structural changes, as confirmed by attenuated total reflectance–Fourier-transform infrared spectroscopy (ATR-FTIR). This was mainly related to an increase in carbonyl-absorbing bands around 1700 cm^−1^ and a decrease in aromatic skeletons near 1500 cm^−1^ in the FTIR spectra, due to the lignin presence (23 wt %) that impacted the colorimetry parameters. Moreover, like the other lignocellulosic materials, cellulose and hemicelluloses remain UV-transparent and degrade more slowly than the lignin fraction, which contains conjugated aromatic structures that absorb strongly in the UV region [17,18].

To resolve this issue, multiple approaches have been investigated in the literature, including those based on the grafting and adsorbing of photostabilizers (UV absorbers and radical scavengers) or even testing surface protection with coatings and chemical modifications of lignocellulosic resources [19,20]. UV absorbers act by absorbing energy and dissipating it as heat while radical scavengers react with the formed radicals, acting as antioxidants. Tragoonwichian and co-workers [21] modified cotton fabrics for improved UV protection based on admicellar polymerization. The authors synthetized 2-hydroxy-4-acryloyloxybenzophenone (HAB) by bonding 2,4-dihydroxybenzophenone to acryloyl chloride. The latter was polymerized on cotton using a sodium dodecylbenzene sulfonate as a surfactant by forming a superficial thin film. The study was evaluated by the authors by use of the Ultraviolet Protection Factor (UPF). The results showed a significant increase in terms of UFP value, from 4 to greater than 40, which highlighted a reduction of UV transmission and an increase in UV protection due to HAB grafting at concentrations above 1.2 mM. In a second work, Tragoonwichian and coworkers [22] copolymerized two UV-absorbing monomers, 2-[3-(2H-benzotriazol-2-yl)-4-hydroxyphenyl]ethyl methacrylate (BEM) and 2-hydroxy-4-acryloyloxybenzophenone (HAB), on cotton fabrics, utilizing admicellar polymerization. The results showed that the fabrics coated using a 1:1 monomer ratio, and with the same total monomer concentration of 3.0 mM for both UV absorbers, yielded the highest UV protection, with a UPF value of 97, in comparison to the 73 and 72 that were obtained from BEM and HAB alone, tested at an equal monomer concentration. An alternative approach was also investigated by the authors of [23]. The authors adopted two-step grafting routes. The first step was based on cotton surface modification using vinyltriethoxysilane to provide polymerizable vinyl groups. In the second step, an ultraviolet-absorbing monomer, 2-[3-(2H-benzotriazol-2-yl)-4-hydroxyphenyl]ethyl methacrylate (BEM), was then polymerized and successfully coated the treated fibers. The study highlighted a significant reduction of ultraviolet transmission, in which UPF values reached 40 and 77 after polymerization using BEM with concentrations of 2.0 mM and 10.0 mM, respectively, in comparison to a UPF of 4 before treatment; this resulted in excellent ultraviolet protection. Gorjanc and coworkers [24] examined the UV-protective properties of untreated and plasma-treated cotton fabric dyed with Cibarcon Deep Red S-B and treated (or not) with Tinofast CEL UV absorber. The investigation showed that UV protection increased after UV-absorber treatment, as quantified by the UPF measurements. The UFP value of the fabrics was about 4 before treatment and increased to 68–74 after treatment. It was also shown that plasma treatment enhanced UV-absorber adsorption, which is linked to an increase in oxygen functional group content on the cotton surface. However, the UV protection seems to decrease after several washing cycles, in which physically attached UV-absorber molecules and dye molecules are removed. This clearly demonstrates the importance of covalent-bond grafting for enhanced UV durability, which is not achieved via surface adsorption only.

Natural fibers’ durability under UV exposure is a critical barrier to their outdoor applications. Moreover, as discussed before, *Miscanthus × giganteus* is of great importance [13], but remains vulnerable to photodegradation, which alters its color and chemical structure. The use of surface coatings or the physical adsorption of UV absorbers lack long-term effectiveness due to weak physical interaction, as opposed to durable covalent bonds. Hence, radiation-induced grafting has recently gained prominence as an effective method used to covalently attach functional molecules to lignocellulosic fibers. This technique, applied as pre-irradiation or simultaneous irradiation, can lead to the generation of free radicals capable of initiating polymerization and covalent-bond formation [25]. Over the years, radiografting has been considered an effective way to modify lignocellulosic materials, and diverse properties such as flame retardancy [8,26], hydrophobicity [27,28], dye and pollutant adsorption [29], carbon dioxide (CO_2_) sorbents [30] and antibacterial applications [31] have been investigated. However, there is a noticeable gap in the literature concerning UV absorbers grafting onto natural fibers, because the radical-scavenging nature of these UV absorbers complicates the process. To the best of our knowledge, no study has systematically evaluated the *e*-beam grafting of UV absorbers onto miscanthus particles.

In this work, we address this gap by investigating both pre-irradiation and simultaneous irradiation techniques used for grafting two different UV absorbers, 2-Hydroxy-4-(methacryloyloxy)benzophenone (HMB) and 2-(4-Benzoyl-3-hydroxyphenoxy) ethyl acrylate (BHEA), onto *Miscanthus × giganteus* particles while avoiding any use of harmful chemicals. These molecules can naturally hinder the formation and stability of the radicals necessary for covalent bonding. Therefore, in the present work, we optimized a variety of factors, including irradiation conditions, solvent systems and additives, to ensure effective grafting. We demonstrated that, while HMB resists grafting, BHEA can be effectively grafted under optimized conditions, leading to improved photostability. We also show that a solvent system and sulfuric acid addition not only increase grafting efficiency but also induce color-related and structural modifications that enhance long-term UV resistance. This efficient grafting and pre-darkening represent a novel strategy for stabilizing natural fibers. By filling this gap, this study aims to provide a novel and environmentally friendly solution, and one which aligns with green chemistry principles by avoiding any use of harmful chemicals, aiming to enhance the UV stability of natural fibers, thereby expanding their potential applications in the composites field and beyond.

## 2. Results and Discussion

**Figure 1:**
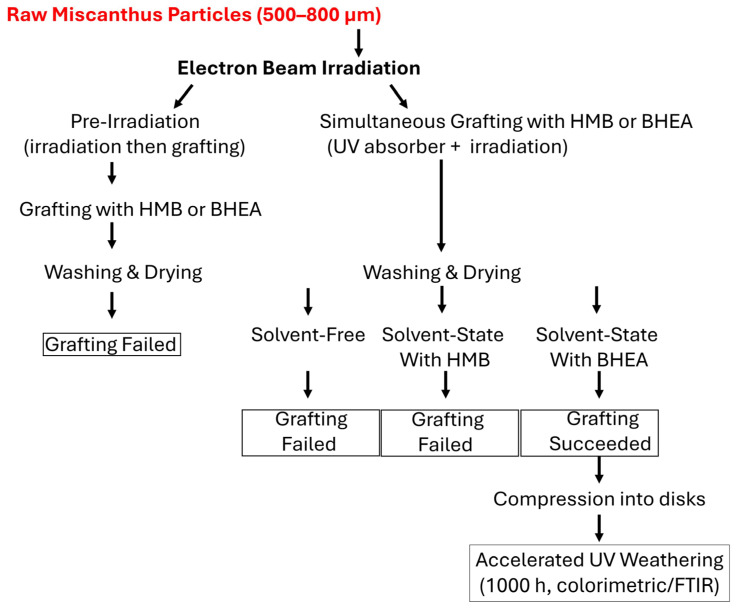
Schematic representation of the grafting routes.

An overview of the experimental pathway of the study is presented as a schematic representation in Figure 1. As shown, Miscanthus particles were subjected to pre-irradiation and simultaneous grafting, followed by the washing and drying of samples before further evaluation. Only the grafted samples were subsequently compressed into disks and subjected to UV-weathering tests for stability assessment. Each grafting route and its outcome is analyzed and discussed in the following sections.

### 2.1. Pre-Irradiation Grafting of HMB and BHEA UV Absorbers

**Figure 2:**
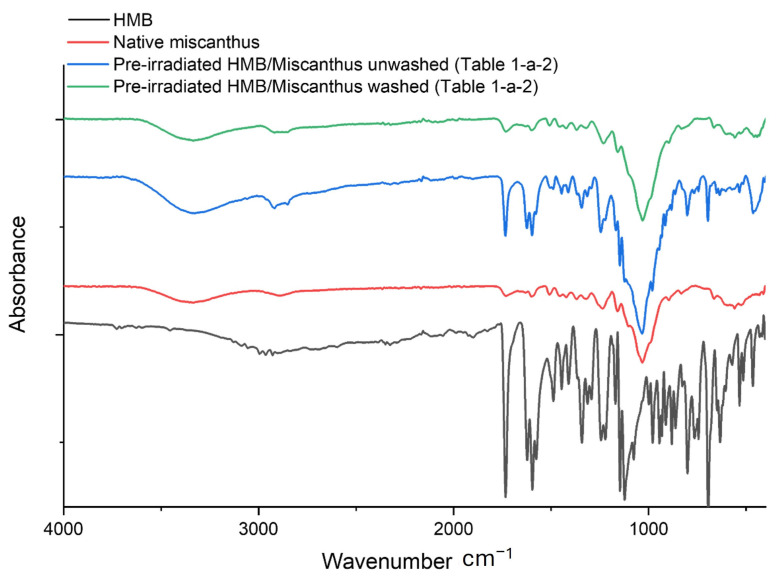
FTIR spectra of HMB, Native miscanthus, Unwashed pre-irradiated HMB/miscanthus and Washed pre-irradiated HMB/miscanthus.

Table 1a shows the results of experiments involving pre-irradiated Miscanthus particles under an air atmosphere. For the grafting step, HMB and BHEA UV absorbers were used in solutions degassed with argon as an inert gas, under various conditions. The most important parameters are the ratio of miscanthus to UV absorber, UV-absorber concentration, butanone/water ratio, ferrous ammonium sulfate Fe(NH_4_)_2_(SO_4_)_2_·6H_2_O (Mohr’s salt) presence, miscanthus/solution concentration, and radiation dose. Radiation generally induces the formation of free radicals throughout the material, which can create active sites and initiate radical copolymerization. The pre-irradiation was performed under an oxygen atmosphere, which forms free radicals capable of initiating the desired chemical reaction, but oxygen could also result in the deactivation of free radicals and the formation of unwanted oxygenated species, such as peroxides. Hence, in the grafting step, argon was used as an inert atmosphere to ensure that free radicals would remain active and free to start the desired grafting reaction, thereby optimizing the efficiency of the grafting process. It is clear from the DG results that no grafting occurred, despite the different UV-absorber ratios (HMB/BHEA), UV-absorber concentrations, and irradiation doses. In the ATR-FTIR spectra (Figure 2), an example of four samples is shown for HMB grafting (the same behavior was observed for BHEA “spectra not shown” and the different grafting conditions used): spectra are drawn for native miscanthus (non-grafted but pre-irradiated at a dose of 50 kGy), HMB molecule, pre-irradiated miscanthus particles after grafting with HMB but before washing (Table 1a-2) and pre-irradiated miscanthus particles after grafting with HMB and washing (Table 1a-2). The native miscanthus spectrum is dominated by broad O–H stretching near 3400 cm^−1^, C–H stretching around 2900 cm^−1^ and characteristic carbohydrate ring vibrations in the 1000–1100 cm^−1^ region [17,32]. Moreover, in the 1700–1500 cm^−1^ region, miscanthus also has characteristic bands due to hemicellulose, cellulose and lignin moieties. The unconjugated C=O stretching of acetyl or ester groups of cellulose and lignin are observed as a sharp band at the 1730–1740 cm^−1^ band. Aromatic C=C skeleton vibrations from lignin components are detected at the 1600 cm^−1^ and 1510 cm^−1^ bands. Since nitrogen content is minimal in miscanthus [33], no strong N–H or C–N features are detected.

In contrast, the spectrum of the aromatic ketone, HMB, displays distinct signals that can include a strong C=O stretch near 1700 cm^−1^ and aromatic-ring vibrations in the 1500–1600 cm^−1^ range. This is typical for conjugated benzophenone ketones [34]. Since no amine groups are present in HMB structure, hence no N–H or C–N bands are expected. It could be observed from the spectrum of pre-irradiated miscanthus particles after grafting reaction but before washing that additional bands were apparent in the 1700–1500 cm^−1^ region that match the UV absorber, indicating that HMB was deposited on the fiber surface. However, once the sample is washed, these HMB-related bands disappear, and the spectrum of the pre-irradiated miscanthus particles after grafting and washing closely resembles that of native miscanthus. This is evident when comparing the 1700–1500 cm^−1^ region, which agrees with the DG results and confirms that the HMB was not covalently grafted and then removed by the washing step.

This behavior could be linked to several factors, such as the ineffective activation of the particles, the chemical properties of HMB and BHEA and improper interaction between the active sites on the miscanthus particles and UV absorbers. It should be highlighted that the ratio of miscanthus to UV absorbers and the concentrations of the UV absorbers did not influence the grafting outcome. Additionally, all experiments with pre-irradiated miscanthus particles were performed at a dose of 50 kGy, except for one experiment conducted at 100 kGy. It is possible that the dose needs to be raised further to cross the threshold of the level needed for effective radical formation and subsequent grafting. A higher dose can also lead to fiber degradation and depolymerization [9]. Mohr’s salt was added in the grafting process, with an aim to improve substrate activation and aid the grafting reaction through the dissociation of peroxides (formed during irradiation in an air atmosphere, as stated before). The level of Mohr’s salt was fixed at a constant 27 mg/g of miscanthus particles across all experiments. Mohr’s salt is utilized to dissociate peroxides present on the miscanthus particles after pre-irradiation [35,36,37]: ROOH + Fe^2+^ → RO^•^ + −OH + Fe^3+^. Nevertheless, the Mohr’s salt addition was unsuccessful.

Additionally, the activation of grafting was conducted in an inert atmosphere, under a controlled argon atmosphere at 80 °C, which is typically used to suppress the oxidation of radicals (residual radicals from irradiation or radicals formed by peroxide decomposition due to the Mohr’s salt). The controlled atmosphere would have favored grafting, yet the absence of grafting reveals that the inert atmosphere alone was not enough to initiate the desired reaction. Moreover, HMB and BHEA molecular structures would not easily be prone to radical grafting onto miscanthus particles. Even though the molecules are UV absorbers, they could be weakly reactive towards radical sites. The study of Brendle and coworkers [6], based on Electron Paramagnetic Resonance analyses, provides explanations for this observation by quantifying the presence of free radicals. Since pre-irradiation of the miscanthus particles was conducted under air, reactive radical species were generated, primarily peroxyl radicals, within the fiber matrix. According to this study, radicals generated in lignocellulosic fibers, such as miscanthus, are largely concentrated in lignin-rich regions. EPR analysis [6] has demonstrated that lignin-based radicals exhibit lower thermal stability compared to those located in cellulose. It seems that at a grafting temperature of 80 °C, the radical species in miscanthus particles decay rapidly, with the majority of the peroxyl radicals disappearing within the first 30 min. This rapid decay indicates that the radical concentration necessary to initiate grafting reactions may not have been present shortly after the start of the experiment. Also, active radicals are required to initiate polymerization, but it seems that their short lifetime at 80 °C prevented the grafting of HMB or BHEA on miscanthus particles.

Brendle and coworkers [8] also proved that the use of Mohr’s salt as a reducing agent permits grafting by dissociation of peroxide species formed during irradiation in air, allowing the initiation of polymerization reaction on lignin-rich substrates, specifically, *Miscanthus × giganteus* stem fragments of dimethyl(methacryloyloxy)methyl phosphonate (MAPC1). Based on that finding, in the present study, once peroxides are dissociated by Mohr’s salt, the resulting alkoxy radicals (RO^•^) can be considered the active species that could initiate the grafting and which could react with the acrylate or methacrylate groups of the grafting agents (HMB and BHEA). Despite that, no grafting occurred, regardless of this parameter, for these UV absorbers, which implies that the presence of Mohr’s salt is not playing an important role in this grafting reaction.

**Figure 3:**
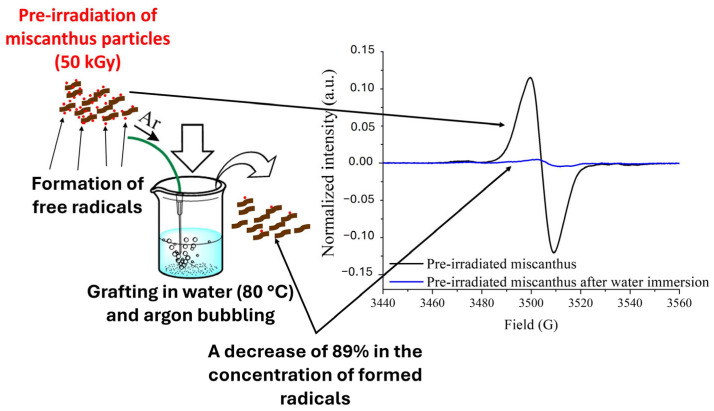
EPR spectra of native pre-irradiated miscanthus particles and post-grafting samples; adapted from Brendle et al. (2014) [8].

In addition, the use of a butanone–water mixture as the solvent seems to have a significant role in the radical dynamics. In fact, the solvent system may influence radical stability on the miscanthus particle’s surface. Grafting will be hindered if the solvent induces radical recombination or quenches radicals. Past research has determined that radical recombination and quenching reactions can be highly influenced by solvents, which consequently influence grafting efficiency directly [35]. According to the EPR results [8], water greatly influences the stability and availability of radicals in lignocellulosic fibers, particularly in miscanthus, due to its highly amorphous and lignin-rich nature compared to flax fibers. When water was added to pre-irradiated miscanthus particles, the radical concentration decreased drastically, keeping only 11% of the initial population of radicals (Figure 3). In this study, the water in the butanone-based solvent system would have caused extensive fiber swelling and increased molecular mobility, enabling water to interact with the radicals. The combination of thermal instability and the water’s quenching of the radicals would have further reduced the radical population, rendering the particles essentially inactive for the initiation of graft polymerization. This quenching effect is consistent with the absence of grafting that was observed in the experiments.

One of the most important parameters that control the outcome is the miscanthus particles’ composition. It was clearly seen in Brendle and co-workers’ [8] work, based on the EPR results, that radicals in lignin are more thermally unstable and more susceptible to interaction with the environmental factors, e.g., water or heat, relative to radicals located in cellulose. Miscanthus particles with high lignin content form radicals of lower stability at elevated temperatures; this was compared by the authors to processes involving cellulose-rich fibers like flax. With the lignin content in miscanthus, radicals formed during pre-irradiation would have been restricted primarily to the lignin matrix. Below 80 °C, these lignin-based radicals undergo decay quite easily, which further explains the rapid loss of radical species during the grafting process.

The absence of grafting in this part of this work can therefore be linked to a variety of factors, i.e., the high level of thermal decay of radicals below 80 °C; the use of a butanone–water solvent system, which led to a quenching effect; and the unstable nature of lignin-based radicals on miscanthus particles. Even though the argon atmosphere prevented oxygen quenching, it was unable to reverse thermal instability and water-induced radical loss.

### 2.2. Solvent-Free Simultaneous-Irradiation Grafting of HMB and BHEA UV Absorbers

**Figure 4:**
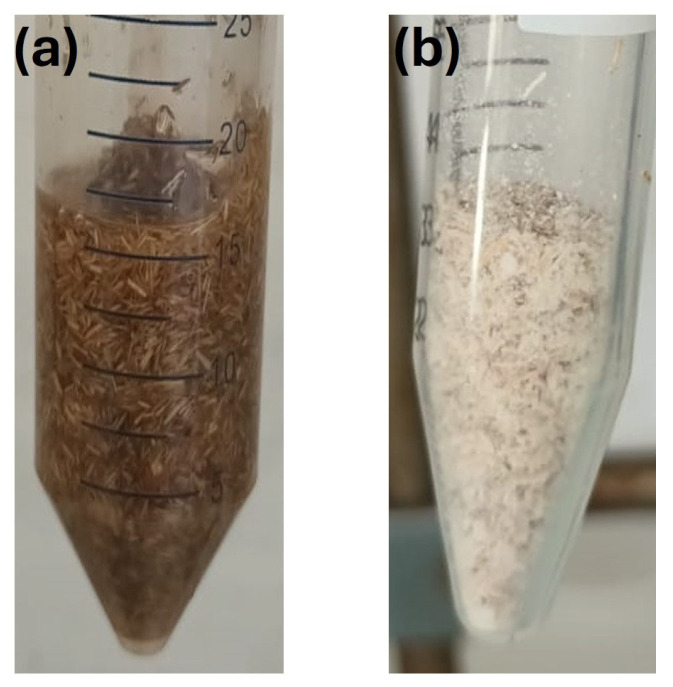
Photographs of miscanthus particles before and after solvent evaporation: (**a**) Particles in solution; (**b**) Particles with BHEA precipitation (white powder) after solvent removal.

The results, shown in Table 1b, of the simultaneous solvent-free grafting reactions of UV absorbers HMB and BHEA onto miscanthus particles via electron beam irradiation under 100 kGy in air showed no detectable grafting (DG% = None), pointing to the main challenges in this approach. FTIR analyses were also performed. The latter results are not presented, since no spectral modifications were detected between native and grafted miscanthus particles, a finding which agrees with the DG results. The failure of grafting can be attributed to the limited mobility of UV absorbers, which form solid residues between the fibers following solvent evaporation, as shown in Figure 4.

In solid-state conditions, the restricted mobility of the UV absorbers would have inhibited their reaction with reactive sites of the particles, a critical factor for successful grafting. In addition, irradiation in the air atmosphere may lead to the quenching by oxygen of the free radicals that initiate the grafting reaction. These results led us to conclude that the lack of observable grafting in the solvent-free simultaneous grafting of UV absorbers HMB and BHEA onto miscanthus particles highlights some of the fundamental difficulties, most significantly the limited mobility of the UV absorbers in their solid-state form.

In contrast, other studies in the literature [8,38] indicate successful examples of grafting where the monomer was in the liquid state, which suggests that mobility of the monomer is crucial in grafting effectiveness. For instance, Brendle and coworkers [26] demonstrated effective grafting of phosphonated monomers on flax fibers when exposed to electron beam irradiation, even with lower doses of irradiation like 10 kGy. The grafting obtained in the study can be attributed in large part to the liquid state of the monomers, which permits efficient interaction with the reactive sites of the samples. Similarly, Hajj and coworkers [38] explored radiation-induced grafting of vinyl phosphonic acid on flax fibers and achieved significant phosphorus grafting using liquid monomer solutions. This suggests that adequate monomer mobility is also required, in addition to factors relating to the radical’s formation, to ensure that the reactive species can access the reactive sites of the particles. Hence, switching to liquid-phase monomers to evaluate the grafting effectiveness of the UV absorbers on miscanthus particles was explored in simultaneous irradiation.

### 2.3. Solvent-Based Simultaneous-Irradiation Grafting of HMB and BHEA UV Absorbers

**Figure 5:**
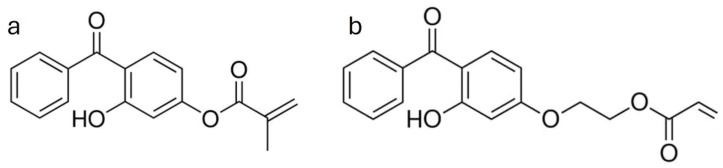
Chemical Structures of (**a**) 2-Hydroxy-4-(methacryloyloxy)benzophenone (HMB) and (**b**) 2-(4-Benzoyl-3-hydroxyphenoxy)ethyl Acrylate (BHEA).

In the experiments summarized in Table 1c, attempts were made to graft 2-Hydroxy-4-(methacryloyloxy) benzophenone (HMB) onto miscanthus particles via simultaneous irradiation under solvent-based conditions. Despite variations in solvent composition (butanone/water), atmosphere (air vs. argon), radiation dose (50–100 kGy) and the presence or absence of Mohr’s salt, no detectable grafting yield was observed in any of the trials. Note that Mohr’s salt was introduced to mitigate the formation of free polymer chains and to limit scenarios in which there is a definitive risk of homo-polymerization [39,40]. FTIR investigations were conducted, but the results are not given, since no spectral differences were detected between native and grafted miscanthus particles, as evidenced by the DG results. One possible explanation might be the resonance stabilization of the methacrylate moiety in the HMB structure (Figure 5), which may reduce the formation of the active radical sites needed for successful grafting. In addition, the benzophenone moiety of HMB can scavenge free radicals in certain instances. The HMB conjugated structure seems to introduce additional stereo and electronic barriers that may hinder efficient grafting [41]. This conjugation induces a delocalization of the radical electron density over the aromatic system and leads to a stabilized intermediate. This stabilizing action reduces the reactivity of the radical species and, therefore, the effective radical concentration available for initiation of grafting reactions [42]. The HMB methacrylate group, while reactive under many polymerization conditions, may be rendered less reactive with the resultant radicals of miscanthus particles, due to this intramolecular resonance. In addition, oxygen availability in an air atmosphere also possesses a proven inhibitive influence relative to radical polymerization [43,44]. Thus, in some experiments (Table 1c-14,15), argon was employed to avoid the inhibitory effect of oxygen. It must be noted that oxygen inhibition is known to be pronounced at lower doses and rates, which was not the case in our experiments [45]. The results indicate that this modification did not promote grafting. Additionally, the irradiation doses and solvent ratios used may not have been the ones most appropriate for grating. Overall, these results suggest that further optimization is necessary to overcome the inhibitory factors and achieve grafting of HMB on miscanthus particles.

**Figure 6:**
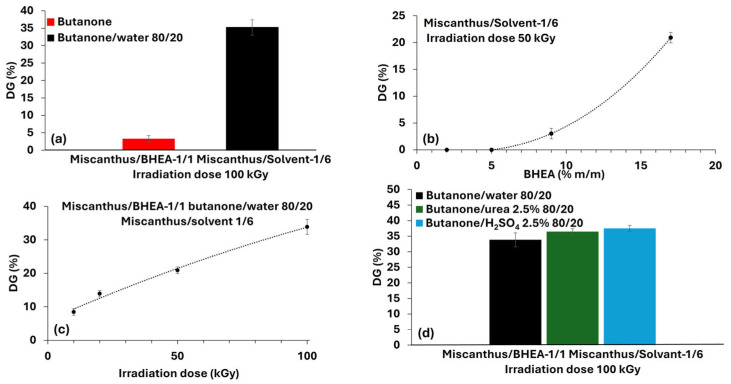
Effects of key parameters on degree of grafting (DG%): influence of (**a**) Solvent composition; (**b**) BHEA concentration; (**c**) Irradiation dose; and (**d**) Additives incorporation.

**Figure 7:**
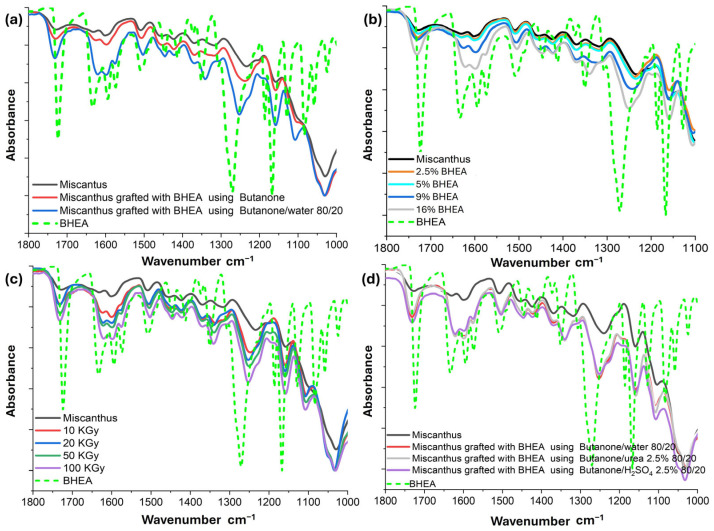
FTIR-ATR spectra of miscanthus particles grafted with 2-(4-benzoyl-3-hydroxyphenoxy) ethyl acrylate (BHEA) under various conditions: (**a**) Solvent effect (butanone vs. butanone/water); (**b**) Effect of BHEA concentration; (**c**) Effect of irradiation dose; and (**d**) Effects of additives (urea and H_2_SO_4_).

To overcome these challenges, one final attempt was made with 2-(4-benzoyl-3-hydroxyphenoxy) ethyl acrylate (BHEA). Relative to HMB, BHEA bears an acrylate moiety and ought to exhibit a diminished resonance effect, considering the longer chain attached to the aromatic ring (Figure 5). This structural difference may reduce both electronic and steric hindrance to grafting [46], hence potentially increasing radical grafting efficiency in miscanthus particles. For that reason, in this section of the work, we studied systematically the parameters influencing the degree of grafting (DG%) of BHEA, based on the solvent-based radiation-induced process. This approach has proven effective, and the BHEA is grafted in varying quantities by the application of certain conditions, which highlights the effects of process parameters on grafting efficiency (Table 1d and Figure 6). The experiments were designed to clarify the roles of important parameters (i.e., the effects of introducing water into butanone, BHEA concentration, irradiation dose and the use of aqueous urea and sulfuric acid as supplementary additives). By comparing grafting efficiencies under these various conditions, we hoped to clarify how each of the parameters influences the graft polymerization. Qualitative Fourier-transform infrared spectroscopy (FTIR) characterization in ATR mode was investigated to evaluate the BHEA grafting by noting increased or additional characteristics evident in the carbonyl and aromatic bands (Figure 7).

#### 2.3.1. Effect of Water Addition

Grafting efficiency is influenced by several parameters, such as solvent composition, fiber swelling and reactive species generation. To study such effects, initial tests were conducted using butanone as the solvent, and then other assays were conducted using an 80/20 (*v*/*v*) butanone/water mixture. Various results were obtained for the two solvent systems (Figure 6a). When using butanone alone, the grafting efficiency remained low, with a grafting yield of only 3.3 ± 1 wt %. When water was added, a significant increase was obtained, reaching 35.2 ± 2 wt %. This observation was confirmed by the FTIR-ATR analysis, as shown in Figure 7a. In this figure the spectra of untreated Miscanthus (irradiated at 100 kGy but without BHEA), of miscanthus grafted with BHEA in pure butanone, of miscanthus grafted with BHEA in a butanone/water (80/20) mixture and of pure BHEA are compared. A key region of interest in FTIR for confirming BHEA grafting is the carbonyl-stretching vibration typically observed around 1720–1730 cm^−1^, which corresponds to the ester group of the acrylate moiety [26,47,48,49]. In addition, aromatic-ring vibrations from the benzoyl/phenolic ring often appear near 1600 cm^−1^ and can become more prominent as the amount of BHEA on the surface increases. For more precision, the pure BHEA spectrum displays strong bands at 1720–1730 cm^−1^ (ester C=O) and near 1600 cm^−1^ (aromatic ring), serving as the reference for identifying graft-related signals. Untreated miscanthus (irradiated at 100 kGy) shows the characteristic lignocellulosic bands, including the broad O–H stretching around 3400 cm^−1^, C–H stretching near 2900 cm^−1^ and various C–O and C–C stretches in the 1050–1150 cm^−1^ region typical of polysaccharides [17,32,50]. Miscanthus grafted in butanone exhibits a moderate variation around 1720–1730 cm^−1^, suggesting the presence of ester groups from BHEA. Some slight enhancements in the aromatic region (around 1600 cm^−1^) are also visible.

**Figure 8:**
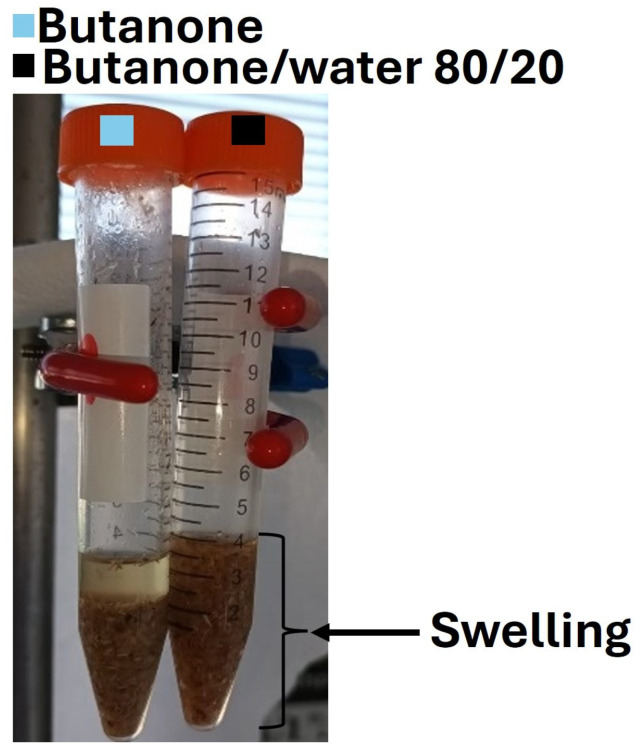
Photograph of miscanthus particles in unmixed butanone vs. butanone/water mixture: evidence of swelling.

However, miscanthus grafted in butanone/water (80/20) exhibits a sharper band in 1720–1730 cm^−1^ and an intense aromatic band close to 1600 cm^−1^, which suggests the higher grafting degree of BHEA [26,47,48,49]. Water is believed to facilitate fiber swelling, enhancing monomer penetration and improving grafting efficiency. These observations confirm that water mixed with butanone improves BHEA grafting, which agrees with the measured degree of grafting (DG). Two mechanisms sems to be behind this improvement. First, water could swell the miscanthus particles and make them more porous, so that the penetration of more monomers can be achieved. This swelling is evident, as observed in the comparative photographs in Figure 8, where the miscanthus particles treated with unmixed butanone remain compact, resulting in two phases with precipitation of fibers. Hence, only surface grafting can be promoted by less-polar solvents, since the monomer diffusion is slower. However, in the presence of a butanone/water mixture, the miscanthus particles swell immensely (Figure 8). Thus, the use of a good swelling solvent like water facilitates bulk grafting by promoting monomer diffusion through the compaction of the miscanthus particles [35]. Such structural alteration is associated with greater accessibility of the reactive sites to grafting. Second, water radiolysis during ionization generates highly active radical species such as hydroxyl radicals (HO^•^), hydrogen radicals (H^•^) and hydroperoxyl radicals (HO_2_^•^), as well as hydrated electrons (aqueous e^−^), among others [29,35,51]. These species enhance local active site density, resulting in more radical species on the miscanthus particles that can permit chain initiation at or near the surface. Thus, the addition of water not only provides greater accessibility to BHEA but might also maximize chemical pathways more suited to favoring grafting and lead to very high levels of improvement in grafting efficiency. Furthermore, water would generate more reactive radicals in comparison to butanone [52], promoting the grafting process even more.

#### 2.3.2. Effect of BHEA Concentration

The impact of varying the BHEA concentration from 2 wt % to 17 wt % was also assessed at 50 kGy, as presented in Table 1d-(2) and Figure 6b. The results show that at very low monomer levels (2 and 5 wt %), grafting was essentially negligible. A moderate grafting of 3.2 ± 1 wt % was obtained at 9 wt % of BHEA, while 17 wt % of BHEA resulted in a high DG of 21.7 ± 2 wt %. FTIR-ATR analysis was also performed, to investigate the effect of BHEA concentration on the grafting of miscanthus particles, as shown in Figure 7b. The spectra include miscanthus alone, samples irradiated at 50 kGy without BHEA and miscanthus grafted with varying BHEA concentrations (2 wt %, 5 wt %, 9 wt % and 17 wt %), alongside pure BHEA as a reference. At low concentrations (2 wt % and 5 wt %), the spectra are like that of native miscanthus, with negligible intensity in the ester carbonyl region 1720–1730 cm^−1^ [8,47,48,49]. This observation aligns with the degree of grafting (DG) values, which were undetectable at these concentrations. However, for 9 wt % BHEA, a slight increase in the carbonyl band and a weak enhancement around 1600 cm^−1^ suggest some degree of grafting, consistent with a measurable but limited DG. At the highest concentration (17 wt %), a significant intensification of the carbonyl band and more distinct aromatic signals near 1600 cm^−1^ confirm a higher grafting level, aligning with the previously reported DG values.

This is explained by the increased availability of monomers at higher concentrations, which resulted in a higher grafting rate [53]. Similar trends have been reported in the literature. For instance, Rasoul and coworkers [54] reported that increases in styrene concentration achieved using two other solvent systems, dichloromethane and benzene, in the grafted medium led to large increases in the extent of the grafting. This outcome was ascribed to the enhancement of monomer diffusivity within the polymer matrix, and the subsequent increase in opportunities for interaction with active sites. Similarly, in the research by Ishak and coworkers [55], the effect of the concentration of GMA, using a water/ethanol mixture, on the yield of grafting was systematically studied in flax textiles. Their findings indicate that it is possible to considerably increase the mass gain directly dependent on the rate of grafting by increasing the concentration of GMA in the grafting solution. For example, when flax fabrics were irradiated at 10 kGy using 1 mol/L GMA solution, the grafting rate was about 15 wt %. When the concentration of GMA was raised to 1.5 mol/L and 2 mol/L, the grafting rates were about 20 wt % and 30 wt %, respectively. These findings are in line with the reported behavior of BHEA in this study, for which the results show that increasing monomer concentration significantly improves grafting efficiency, indicting the necessity of optimizing the monomer concentrations in simultaneous radiation grafting processes.

#### 2.3.3. Effect of Irradiation Dose

The effect of various irradiation doses on the degree of grafting (DG) of BHEA in miscanthus particles was studied. The amount of BHEA was kept constant at 17 wt %, a value already established as being optimal for the achievement of high grafting levels. The irradiation doses employed ranged from 10 to 100 kGy. As shown in Table 1d-(3) and Figure 6c, varying the irradiation dose led to a progressive rise in grafting: 8.7 ± 1 wt % at 10 kGy, 14.4 ± 1 wt % at 20 kGy, 21.7 ± 2 wt % at 50 kGy and 35.2 ± 2 wt % at 100 kGy. The results demonstrate an almost linear trend of increasing DG relative to higher irradiation doses, indicating that radiation dose is a critical parameter in the grafting process of BHEA. The FTIR-ATR spectra, as shown in Figure 7c, for particles irradiated under different irradiation doses display a parallel intensification of the key BHEA bands. It can be observed that progressive modifications of miscanthus spectrum are obtained with increases in the irradiation dose. While still qualitative, these spectral observations clearly substantiate the determination that higher doses produce more grafted polymer chains on the substrate. Importantly, the appearance and gradual intensification of the band near 1720 cm^−1^, attributed to the ester C=O stretching of BHEA, strongly support successful grafting [8,47,48,49]. This band, barely visible at 10 kGy, becomes increasingly intense at higher doses (20, 50 and 100 kGy), confirming the progressive incorporation of BHEA as the degree of grafting (DG) rises, as quantified previously. Furthermore, the intensification of aromatic C=C stretching bands at 1600 and 1510 cm^−1^, not prominent in raw miscanthus but characteristic of BHEA, corroborates the grafting. These bands also increase in intensity with higher doses, in agreement with the finding of increased DG. Additionally, modifications are observed in the C–O–C and C–O bands within the 1250–1000 cm^−1^ regions, where both lignocellulosic components and ester groups of BHEA absorb. The increased complexity and intensification in this region suggest overlapping contributions from both grafted BHEA moieties and miscanthus particles. Hence, the increases in the intensity of the ester (C=O) and aromatic (C=C) bands directly reflect the amount of BHEA grafted, highlighting the role of irradiation in promoting the radical sites required for efficient graft polymerization [56].

The progressive rise in DG can be attributed to the formation of a greater number of radical sites on the miscanthus particles as the radiation dose increases. Higher doses of irradiation generate more free radicals, which then serve as active sites for the initiation of graft polymerization [35]. This dose–radical-formation relationship is known in the literature. For instance, in research investigating the radiation grafting of poly(butyl acrylate) on ethylene vinyl acetate copolymer, it was observed that an increase in the gamma irradiation dose from 10 to 50 kGy led to a concurrent increase in the grafting yield because of the higher availability of high-energy radiation for the formation of active sites for monomer grafting [56]. The same tendency has been reported elsewhere in the literature. Ishak and coworkers [55] investigated the radiation-induced grafting of glycidyl methacrylate on flax fabrics and reported that the grafting percentage increased with increased doses of irradiation. Also, Rasoul and co-workers [54] investigated the radiografting of styrene onto (poly(tetrafluoroethylene-co-ethylene) and reported the linear dependence of the radiation dose on grafting efficiency at an optimum level. These findings point out the importance of irradiation dose as a controlling factor, even for BHEA, in the context of miscanthus particles. Hence, the higher availability of radicals induced by higher doses allows for more efficient attachment of monomers, improving the overall grafting yield.

#### 2.3.4. Effects of the Addition of Additives (Urea and H_2_SO_4_)

In the final set of trials (Figure 6d), the water portion in the 80/20 butanone/water system was replaced by either 2.5 wt % aqueous urea or 2.5 wt % aqueous H_2_SO_4_. Both additives led to modest yet measurable gains in DG relative to the standard 80/20 mixture: 36.6 ± 1 wt % (urea) and 38.4 ± 1 wt % (H_2_SO_4_), versus 35.2 ± 2 wt % without additives. Urea can increase fiber swelling and solubilize some fiber components, thereby enhancing monomer penetration [57,58]. However, H_2_SO_4_ might trigger further radical-formation mechanisms, possibly through acid-catalyzed reactions in the fiber surface or through a modified radiolysis process. The presence of H^+^ ions is crucial in inducing the conversion of solvated electrons to H^•^ radicals, which induce radical formation on the substrate through hydrogen atom abstraction [59,60,61]. Therefore, the addition of H_2_SO_4_ can slightly enhance grafting yields in some systems. Thus, in this study, both additives contribute to a limited increase in grafting yield. These small differences are not reflected in the FTIR spectra of treated samples (Figure 7d).

### 2.4. Accelerated UV Weathering of Compressed Miscanthus-Based Discs

**Figure 9:**
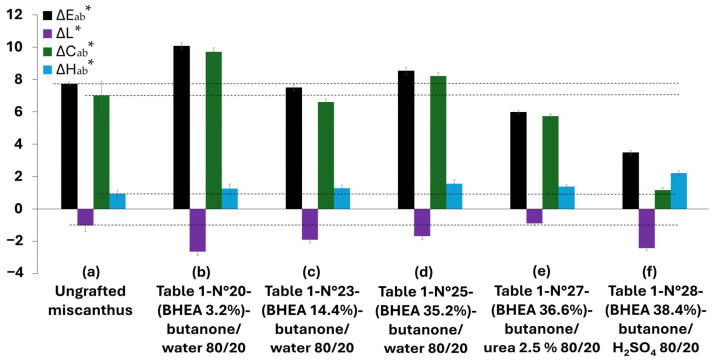
Color variation (∆E_ab_*), lightness (∆L*), chroma (∆C_ab_*) and hue (∆H_ab_*) of raw miscanthus (**a**) ungrafted and miscanthus grafted with different concentrations of BHEA [(**b**) 3.2%, (**c**) 14.4 %, (**d**) 35.2%, (**e**) 36.6% and (**f**) 38.4%], after 1000 h of UV weathering. (Dashed curves are included as guidelines for following the evolution of each color component in the results.).

The photostability of lignocellulosic materials is a complex phenomenon influenced by their intrinsic chemical composition, the presence of chromophoric groups and the modifications induced by grafting treatments [17,19,21,22,23,24]. In this study, the evolution in the color of miscanthus-based discs after 1000 h of UV weathering was assessed using four key parameters: total-color variation ∆E_ab_*, lightness (∆L*), chroma (∆C_ab_*) and hue (∆H_ab_*). These parameters provide complementary information regarding the overall color change ∆E_ab_*. Figure 9 presents the results for the color change parameters, in diagram form. It should be noted that samples exhibiting detectable modifications in terms of effective and progressive grafting were retained for further UV weathering and characterization.

For the ungrafted miscanthus, UV exposure for 1000 h resulted in a moderate overall color change (∆E_ab_* ≈ 7.7) that exceeded the threshold value of 2, the limit at which the human eye perceives a color difference, and surpassed 5, the threshold value at which colors are distinctly recognized as different [62]. The results show also a slight darkening (∆L* ≈ –1.02), a marked increase in chroma (∆C_ab_* ≈ 7), and only minimal hue shift (∆H_ab_* ≈ 0.94). These observations align with our previous work [17], in which, owing to the relatively high lignin content (≈23%) of the samples, the development of chromophoric carbonyl and quinonoid structures under UV irradiation led primarily to browning and increased color saturation.

Grafting treatments using BHEA in various formulations were explored to evaluate the photostability of miscanthus-based discs. The results show that none of the treatments (Table 1, N° 20, 23, 25 and 27) provided a satisfactory improvement in UV photostability, as they all produced overall color changes (∆E_ab_*) above the perceptibility threshold of 5, indicating that the treated miscanthus particles exhibited distinct color differences that could be clearly recognized by the human eye. For instance, miscanthus particles treated with a low BHEA level (3.2 wt % in a butanone/water system) showed a high color variation (∆E_ab_* ≈ 10.1), stronger darkening (∆L* ≈ –2.6), a marked increase in chroma (∆C_ab_* ≈ 9.7) and a modest hue shift (∆H_ab_* ≈ 1.3). This would mean that limited grafting not only fails to protect the fibers, but may also add more reactive sites, which contribute more to photodegradation and lead to greater color changes. With the grafting level increased to 14.4 wt % in the same butanone/water system, the behavior of the samples began to resemble that of the ungrafted miscanthus particles, exhibiting a less pronounced total-color change (∆E_ab_* ≈ 7.5) and chroma change (∆C_ab_* ≈ 6.5), while darkening (∆L* ≈ –2.5) and hue shift (∆H_ab_* ≈ 1.3) remained significant. This indicates that, while a higher grafting level may help to limit the formation of additional chromophoric groups, it does not effectively counteract the oxidative degradation that leads to color changes. With 35.2 wt % BHEA grafting, the total-color variation rebounded to around ∆E_ab_* ≈ 9, with persistent darkening (∆L* ≈ –2.7) and further increases in chroma (∆C_ab_* ≈ 8.5) and hue (∆H_ab_* ≈ 1.6). This suggests that an elevated grafting ratio within a water-based system can be responsible for the accumulation of secondary chromophore structures, ruining the potential benefits of surface modification. Similarly, increasing the grafting level to 36.6 wt % using a butanone/urea system provided only a partial decrease in color change (∆E_ab_* ≈ 6.0), slight darkening (∆L* ≈ –0.9) and moderate increases in chroma (∆C_ab_* ≈ 5.7) and hue (∆H_ab_* ≈ 1.4). Although this modification resulted in a slightly lower level of total-color variation, the variation still exceeded the perceptibility threshold, reinforcing the determination that increasing the degree of grafting does not necessarily improve UV stability.

**Figure 10:**
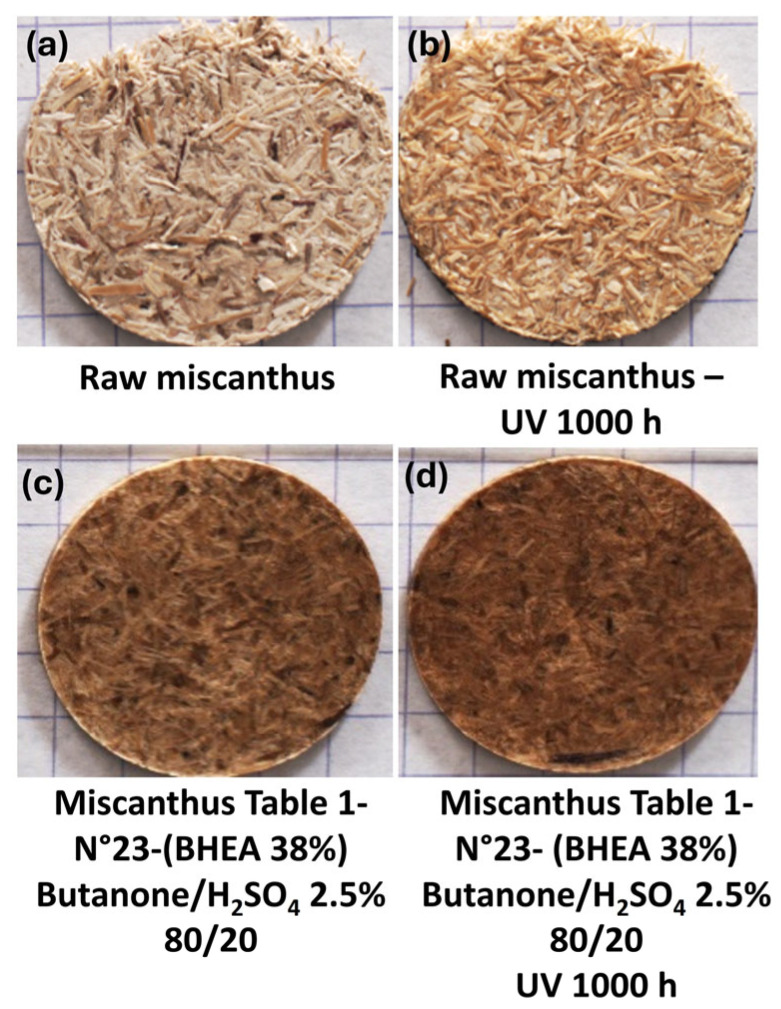
Photographs of raw miscanthus (**a**) before and (**b**) after 1000 h of weathering and miscanthus grafted with 38% of BHEA (Table 1, N° 23) discs, (**c**) before and (**d**) after 1000 h of weathering.

The most significant enhancement in UV stability was achieved using a formulation of 38.4 wt % BHEA with 2.5% sulfuric acid in a butanone system. This treatment yielded the lowest overall color change (∆E_ab_* ≈ 3.4) and the smallest variation in chroma (∆C_ab_* ≈ 1.2). Although this formulation exhibited a high degree of darkening (∆L* ≈ −2.4) and the highest hue (∆H_ab_* ≈ 2.2), the overall perceptible color change was minimized. This could be directly linked to the color modification in the miscanthus fibers after treatment and before UV weathering, as can be clearly observed in Figure 10a compared to Figure 10c.

**Figure 11:**
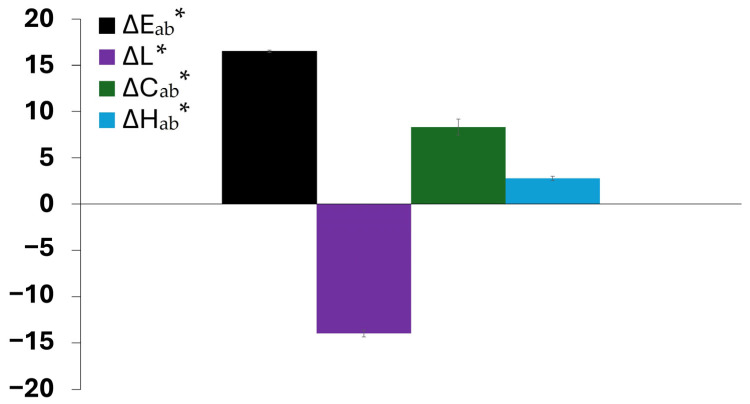
Total-color variation (∆E_ab_*), lightness (∆L*), chroma (∆C_ab_*) and hue (∆H_ab_*) of miscanthus grafted with BHEA in butanone/2.5% H_2_SO_4_ 80/20 system before weathering.

**Figure 12:**
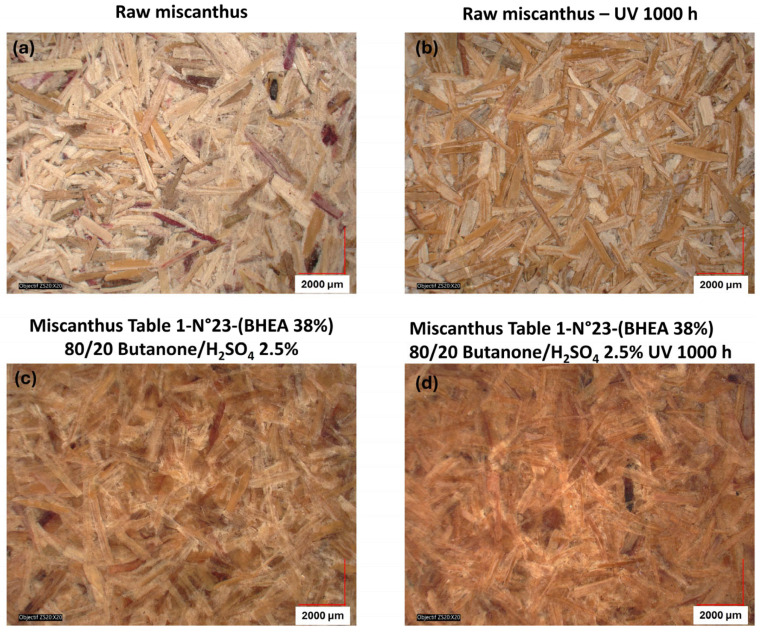
Microscopic observations of raw miscanthus discs (**a**) before and (**b**) after 1000 h of UV weathering and miscanthus grafted 38% of BHEA (**c**) before and (**d**) after 1000 h of UV weathering (Table 1, N° 23), before and after 1000 h of UV weathering.

For aid of understanding, Figure 11 presents the color variation measurements for miscanthus particles grafted in acidic medium before exposure to weathering (Figure 10c). The calculation of the color parameters was made against untreated miscanthus fibers (Figure 10a). The results show a significant increase in overall color change (∆E_ab_* ≈ 16.5). Such a high increase points out that the acid treatment plays an important part in changing the natural color of fibers even before their exposure to UV. The most remarkable observation is the strong decrease in lightness (∆L* ≈ −14), showing a darkening effect favored by the grafting reaction in acid medium. This tendency is coupled with chroma (∆C_ab_* ≈ 8.0) and hue (∆H_ab_* ≈ 2.8) changes that together are responsible for the perceptible color shift after grafting and before weathering. The darkening of the fibers before weathering is representative of a structural or chemical change in the lignocellulosic components, which can be a precursor to improved UV stability. This observation agrees with the work of Shi and coworkers [63] on sulfuric acid treatment of wood; the work suggests that wood treatment with sulfuric acid could lead to a perceptible color shift in which the color turned to a dark chocolate-brown because of the acid degradation before UV exposure. Acidic treatment likely promotes conjugated chromophore development via glycosidic linkage cleavage and modification of aromatic-ring structures in lignin. This effect is in line with the dominant darkening effect (∆L* ≈ −14) found in this study and suggests that new chromophore structures enhance visible light absorption. Hence, the impact of this initial color modification becomes evident when examining the long-term weathering behavior, as shown in Figure 9 and Figure 10. In the case of native (untreated) miscanthus, UV exposure over 1000 h led to a significant color change, with visible browning and particle decohesion, as confirmed by the microscopic observations in Figure 12b. As previously discussed, this browning is attributed to lignin degradation leading to the formation of chromophore carbonyl and quinoid structures, which are responsible for color saturation and an increase in darkening [17]. Contrarily, the miscanthus particles grafted with 38 wt % of BHEA in acidic medium exhibited a steady post-weathering color profile, as seen in Figure 9 and Figure 10d.

The pre-weathering darkening effect appears to have positively influenced the fiber’s resistance to UV-induced color modification, as the overall post-weathering color change was minimized. Hence, BHEA-grafted miscanthus maintains a more intact fiber structure and a cohesive miscanthus-based disc with fewer visible defects and a more homogeneous color distribution, as shown by the microscopic observations in Figure 12d. This indicates that the chemical modification not only suppresses color alteration but also ensures the physical integrity of the particles, hindering excessive degradation. This suggests that acid-catalyzed grafting not only alters the color of the particles initially, but would also enhance resistance to photodegradation.

These findings underscore the significance of chemical modification methods in controlling miscanthus particles’ photodegradation. While acid treatment alters the inherent color, it becomes a promising strategy that ensures increased color stability. Rather than having an inherent color that tends to depreciate with time, color darkening is more suitable for ensuring the increased UV stability of miscanthus particles through minimizing the perceived color change.

## 3. Materials and Methods

### 3.1. Materials

Miscanthus raw materials were harvested in Normandy, France, and conditioned in sealed plastic bags, which were produced by Addiplast company (Saint-Pal-de-Mons, France). Miscanthus particles underwent a sieving process to remove any dusty components and retain fragments within the 500–800 µm size range. Ethanol (96% purity) was procured from PANREAC (Castellar del Vallès, Spain). Sulfuric acid (98% purity) was obtained from CHEM-LAB (Zedelgem, Belgium). Tetrahydrofuran (99.8% purity) and Butanone (99% purity) were purchased from Fisher Scientific (Illkirch, France). Mohr’s salt Prolabo ((NH_4_)_2_Fe (SO_4_)_2_, 6 H_2_O) was supplied by VWR (Les Ulis, France). 2-Hydroxy-4-(methacryloyloxy)benzophenone (HMB, 99% purity, Figure 5a) and 2-(4-Benzoyl-3-hydroxyphenoxy)ethyl acrylate (BHEA, 97% purity, Figure 5b) UV absorbers were acquired from ABCR (Lyon, France).

### 3.2. Methods

#### 3.2.1. General Procedure of Miscanthus Particle Modification by Pre-Irradiation Grafting

Miscanthus particles were irradiated at Ionisos SA (Chaumesnil, France), using a Linac electron beam (*e*-beam) delivering a dose rate of approximately 300 kGy/min and operating at 10 MeV and 34 kW. The target doses, measured by polystyrene calorimetry (uncertainty = 3.8%), were set at 50 kGy and 100 kGy, with the process carried out in air at room temperature. To prevent rises in the temperature of the irradiated samples, the 50 kGy and 100 kGy doses were delivered in two and four passes, respectively, each limited to 25 kGy per pass. The irradiated miscanthus particles were immediately stored at −18 °C and transported to the laboratory in refrigerated containers at the same temperature, to ensure the stability of the generated radical species. Throughout the study, the irradiated particles were stored in a freezer at −15 °C and removed only a few minutes before the grafting tests. HMB and BHEA solutions were prepared in butanone, as this solvent offers the highest solubility for these molecules, with concentrations reaching up to 17 wt % in butanone. In contrast, the solubility of these molecules in water is significantly limited; hence, the water content in the butanone mixture was minimized. To achieve the grafting process, the HMB and BHEA prepared solutions were degassed by argon, as an inert gas, for 15 min in a container before the addition of the pre-irradiated miscanthus particles. In most of the assays, Mohr’s salt was added to the aqueous part before bubbling. The conditions tested involved varying the following parameters: Miscanthus/BHEA or HMB mass ratios; butanone/water mass ratios; BHEA concentration; and miscanthus/solution mass ratios. Table 1a summarizes the different assays prepared in this part. After the pouring of the pre-irradiated miscanthus particles, degassing continued for an additional 2 min, and the centrifuge tubes were each tightly closed with a cap. The containers were then heated to 80 °C for 1 h. The treated miscanthus particles were recovered through filtration and washed 3 times with butanone and distilled water, each wash conducted at 40 °C for 10 min in an ultrasonic bath. The particles were allowed to dry in a fume hood for 24 h until weight stabilization. For the purpose of accuracy, each experiment was conducted in duplicate.

#### 3.2.2. General Procedure of Miscanthus Particle Modification by Simultaneous-Irradiation Grafting

Simultaneous irradiation grafting was achieved via 2 routes. The first route is based on solvent-free grafting during irradiation and adapted from the work of Hajj et al. (2019) [38]. Hence, miscanthus particles were mixed with HMB dissolved in butanone; the particle/solution weight ratio was 1/1. After mixing, the particles were allowed to embed in the solution for 1 h. The mixture was then placed in an extractor hood to facilitate solvent evaporation. The dried miscanthus particles were irradiated with the *e*-beam at a radiation dose of 100 kGy, in air and at room temperature, by Ionisos SA (Chaumesnil, France). Once received, the particles were washed 3 times with 40 °C butanone in an ultrasonic bath to eliminate any unreacted monomers, oligomers, or polymers. The washed particles were then left in an extractor hood to dry until their weights stabilized. To ensure precision, the experiment was performed in triplicate. The various assays of this route are summarized in Table 1b. The second route involves grafting in a solvent state during irradiation, inspired by the method described by Ishak et al. (2024) [55]. HMB and BHEA were dissolved in butanone or an 80/20 butanone/water mixture, then combined with miscanthus particles in a centrifuge tube, which was immediately sealed to prevent solvent evaporation. The centrifuge tubes containing both the particles and the UV absorbers dissolved in the solvent were exposed to *e*-beam irradiation at different radiation doses, in air and at room temperature; this was realized by Ionisos SA (Chaumesnil, France). It is important to note that only the miscanthus/BHEA and miscanthus/solution ratios were assessed. Additionally, some tests were conducted replacing the water part with other additives, including sulfuric acid (2.5 wt % H_2_SO_4_) and urea (2.5 wt %). The irradiated samples were filtered; washed 3 times with butanone/water 80/20 and two times with butanone, at 40 °C in an ultrasonic bath; and then dried in an oven at 60 °C for 2 h and then left under the fume hood until weight stabilization. To ensure the validity of the results, each experiment was repeated in triplicate. An overview of the various assays of this route is presented in Table 1c,d.

#### 3.2.3. Miscanthus Disc Preparation

Following the method outlined by El Hage and co-workers [17], binder-less compressed discs of miscanthus were prepared by measuring 500 mg of raw or modified particles. These particles were then compressed under a pressure of 30 kN for 2 min at a temperature of 105 °C, utilizing a Prontopress-2 press (Struers A/S, Copenhagen, Denmark). The resulting discs, each with a diameter of 2.5 cm and a thickness of 2 mm, were allowed to cool to room temperature for 4 min before being recovered.

#### 3.2.4. Accelerated UV Weathering

Two discs of each miscanthus sample were placed in a QUV/se accelerated weathering tester (Q-Lab, Cleveland, OH, USA). Aging was conducted, following the condition described by El Hage et al. (2024) [17], for a total of 1000 h. Repetitive 12-hour cycles were applied, consisting of 8 h of UV exposure at a wavelength of 340 nm, with an irradiance of 0.76 W/m^2^ and a temperature of 60 °C, followed by 4 h of dark exposure at 40 °C. After a total of 1000 h of UV exposure, the samples were recovered for evaluation and analysis.

#### 3.2.5. Spectrophotometer Analysis

Following the experimental procedure outlined by El Hage and coworkers [17], color measurements of the miscanthus compressed discs, both before and after weathering, were conducted using a polychromator-type spectroradiometer (Konica-Minolta CS-2000, Tokyo, Japan). This instrument enables precise light measurements, allowing for the determination of the spectral reflectance of colored surfaces. The CIE L*a*b* 1976 values were then calculated, along with chroma (C*), which measures the intensity of a color, and hue angle (h*), which determines a color’s position on the color wheel. These parameters were determined by averaging three measurements taken for each area. The total-color difference ($∆E_{ab}^{*}=\sqrt{{∆L}^{2}+{∆a}^{2}+{∆b}^{2}})$, the lightness (∆L*), the chroma (∆C_ab_*) and the hue (∆H_ab_*) were calculated and compared. ∆a and ∆b, which are parts of the calculation for ∆E_ab_*, denote the differences in the a* and b* components between two colors, reflecting shifts in the green–red and blue–yellow hues, respectively. An increase in ∆L* indicates a lightening, while a decrease indicates a darkening of the color. ∆C_ab_* measures the change in chroma, with higher values indicating increased saturation. ∆H_ab_* represents the difference in hue angles between the colors.

#### 3.2.6. Attenuated Total Reflectance–Fourier-Transform Infrared Spectroscopy (ATR-FTIR)

The surfaces of both native and grafted miscanthus particle discs were analyzed before and after grafting, using a Vertex 70 single-reflection diamond ATR-FTIR spectrometer (Bruker Corporation, Ettlingen, Germany). For each sample, baseline-corrected spectra were recorded at different exposure times, with 32 scans and a resolution of 4 cm^−1^. FTIR signals and the corresponding assignments for *Miscanthus × giganteus* particles are compiled in Supporting Information S1.

#### 3.2.7. Optical Microscope Observation

The surfaces of the raw and grafted miscanthus particle disks were observed using a fifth generation Keyence VHX-7000 digital microscope (Itasca, IL, USA). Images were captured with a high-performance VHX-7020 camera, utilizing the VH-ZST real zoom lens, at a standard overall magnification of 100x.

#### 3.2.8. Degree of Grafting Measurements (DG wt %)

For all grafting procedures, the degree of grafting was assessed by weighing the particles with an Quintix124-1 Sartorius^®^ analytical balance (Goettingen, Germany), with a precision of 10^−4^ g. Particle weights were recorded both before and after grafting. The dry mass of the particles was determined using a Sartorius™ MA 160 infrared moisture analyzer (Goettingen, Germany). In this procedure, 0.2 g of particles are heated to 105 °C until completely dried. The dry matter percentage is automatically calculated by the infrared balance, accounting for the initial and final fiber masses. Each analysis was performed in triplicate to ensure reproducibility, and the average value from these repetitions was used. An additional parameter was considered to account for the weight loss that could occur from the washing steps, due to the use of solvents that remove extractive compounds. To address this, identical experiments were conducted without UV absorbers while applying the same washing procedures. Consequently, the weight loss fraction (P) associated with these washing steps was estimated and included in the calculation to avoid any underestimation in the calculation of the grafting degree. Hence, the degree of grafting is calculated as follows in Equation (1):

(1) $$DG\;wt\;\%=\left[100\cdot \frac{\left(final\;dry\;weight-starting\;dry\;weight\times (1-P)\right)\left(g\right)}{starting\;dry\;weight\times (1-P)\left(g\right)}\right]$$

## 4. Conclusions

This work highlights the need for UV-absorber grafting onto *Miscanthus × giganteus* particles to alleviate the inherent tendency of these particles towards photodegradation. The pre-irradiation grafting attempts with HMB and BHEA were failed attempts, mainly due to the rapid radical degradation under the tested conditions. Solvent-free simultaneous grafting was also unsuccessful as the solid-state UV absorbers lacked the mobility necessary for penetration of the particles and initiation of covalent bonding. Simultaneous irradiation of HMB within a solvent system was also unable to yield detectable grafting, likely due to the HMB’s radical-scavenging and resonance stabilization ability, which inhibited polymerization. On the other hand, simultaneous irradiation under butanone/water conditions, and especially with BHEA, led to effective grafting and greatly improved the UV stability. Among the significant parameters influencing graft yield were solvent composition, BHEA concentration, irradiation dose and the incorporation of additives such as urea or sulfuric acid. Most notably, the highest grafting level (38 wt %) and photoprotection were achieved by grafting BHEA in a butanone medium containing 2.5% sulfuric acid. Although this formulation produced a minimized overall color change (∆E_ab_* ≈ 3.4 after 1000 h of accelerated UV exposure), the enhanced photostability was not directly linked to the degree of grafting but rather to a pronounced initial darkening of the fibers induced by the sulfuric acid treatment prior to weathering. This preliminary color transition appears to alter the chromophore structure of the particles and consequently partially inhibit subsequent UV alteration. These findings validate successful miscanthus grafting through BHEA monomer and propose a complex inter-relationship between color stability, chemical modification and BHEA grafting. Hence, beyond grafting efficiency, the initial chemical treatment plays a crucial role in defining the long-term photostability of the material. Additional studies could be focused on the incorporation of these fibers in a polymeric matrix, and a scale-up of the process for industrial applications in the fiber-reinforced composites field, to evaluate their durability.

## Figures and Tables

**Table 1:** Experimental conditions and degree of grafting (DG, wt %), for pre-irradiation and simultaneous-irradiation routes: (**a**) pre-irradiation in air atmosphere followed by grafting in argon atmosphere (HMB or BHEA); (**b**) simultaneous solvent-free (HMB or BHEA); (**c**) Simultaneous in solvent state (HMB); and (**d**) simultaneous in solvent state (BEHA).

N°	Miscanthus/HMB or BHEA (m/m)	UV Absorber (wt %)	Butanone/Water (m/m)	Mohr’s Salt/Miscanthus (mg/g)	Miscanthus/Solvent (m/m)	Atmosphere	Dose (kGy)	DG (wt %)
**(a) Pre-irradiation** **under air atmosphere with grafting in argon atmosphere (HMB or BHEA)**								
1	1/1	5	80/20	-	1/20	Argon	50	None
2	1/1.5	8	90/20	27	1/20	Argon	50	None
3	1/1.5	8	95/5	27	1/20	Argon	50	None
4	1/1.5	8	90/10	27	1/20	Argon	50	None
5	1/1.5	8	80/20	27	1/20	Argon	50	None
6	1/1.5	8	70/30	27	1/20	Argon	50	None
7	1/1.5	8	90/10	27	1/20	Argon	100	None
**(b) Simultaneous solvent-free (HMB or BHEA)**								
8	1/1	-	-	-	-	Air	50	None
9	1/1	-	-	-	-	Air	100	None
**(c) Simultaneous in solvent state (HMB)**								
10	1/1	11	100	-	1/9	Air	50	None
11	1/1	6	100	-	1/18	Air	50	None
12	1/1	13	80/20	27	1/8	Air	100	None
13	1/1.5	15	80/20	-	1/10	Air	100	None
14	1/1	6	80/20	-	1/18	Argon	100	None
15	1/1	11	100	-	1/9	Argon	100	None
**(d) Simultaneous in solvent state (BEHA)**								
**(1) Effect of water addition**								
16	1/1	17	100	-	1/6	Air	100	3.3 ± 1
17	1/1	17	80/20	-	1/6	Air	100	35.2 ± 2
**(2) Effect of BHEA concentration**								
18	1/0.125	2	80/20	-	1/6	Air	50	None
19	1/0.25	5	80/20	-	1/6	Air	50	None
20	1/0.5	9	80/20	-	1/6	Air	50	3.2 ± 1
21	1/1	17	80/20	-	1/6	Air	50	21.7 ± 2
**(3) Effect of irradiation dose**								
22	1/1	17	80/20	-	1/6	Air	10	8.7 ± 1
23	1/1	17	80/20	-	1/6	Air	20	14.4 ± 1
24	1/1	17	80/20	-	1/6	Air	50	21.7 ± 2
25	1/1	17	80/20	-	1/6	Air	100	35.2 ± 2
**(4) Effect of additives addition**								
26	1/1	17	80/20	-	1/6	Air	100	35.2 ± 2
27	1/1	17	80/20 *	-	1/6	Air	100	36.6 ± 1
28	1/1	17	80/20 **	-	1/6	Air	100	38.4 ± 1

* 80/20: the water was replaced by 2.5 wt % aqueous urea solution. ** 80/20: the water was replaced by 2.5 wt % aqueous sulfuric acid solution (H_2_SO_4_).

## Data Availability

Data is contained within the article.

## Supplementary Materials

The following supporting information can be downloaded at: https://www.mdpi.com/article/10.3390/molecules30173649/s1, Supporting Information S1: FTIR signals and corresponding assignment for *Miscanthus × giganteus* particles in this study.
