# Supplementary material for: Evaluating UV Stability of Miscanthus × giganteus Particles via Radiografting of UV Absorbers
Source: Molecules. 2025 Sep 8;30(17):3649. doi: 10.3390/molecules30173649 (PMC12430285; doi:10.3390/molecules30173649)
Supplement: Supplementary file 1 [file molecules-30-03649-s001.zip › molecules-3774446-supplementary.pdf]

Supporting Information 1:

FTIR signals and corresponding assignment for miscanthus x giganteus particles in this study

Wave number (cm^{-1})	Functional group	Assignment	Moieties
3324	O-H	Stretching	Cellulose, hemicellulose, lignin, and extractives
2917	CH_2	Asymmetric stretching	Extractives
2850	CH_3	Symmetrical vibration	Extractives
2895	C-H or CH_2	Stretching	Cellulose, hemicellulose, and lignin
1730	C=O	Stretching	Mainly for Hemicellulose (weakly for lignin and extractives)
1640	C=O	Stretching	Lignin (weakly)
	H-O-H	Deformation	Absorbed water in cellulose and hemicellulose
1600 and 1510	C=C	Stretching	Mainly for lignin and
1456	C-H	Deformation	weakly for extractives
1421 and 1370	H-C-H / O-C-H	Bending vibration	Cellulose
	C-H (in CH_3 and phenol)	Deformation	Lignin and extractives
1429	H-C-H	Bending vibration	Cellulose
1317	CH_2	Rocking vibration	Mainly cellulose
	C-O	Stretching	Lignin (Weakly)
1240	C-O	Stretching	Lignin, cellulose, and Hemicellulose
1159	C-O-C	Asymmetric stretching	Cellulose and hemicellulose
	C-H	Stretching	Extractives and lignin
1103, 1029, 985	C-C, C-OH, C-H	Vibration	Cellulose and hemicellulose
833	C-H	Out-of-plan deformation	Lignin
663	C-OH	Out-of-plan bending	Cellulose
558			
